# Benzyl­hexa­decyl­dimethyl­ammonium chloride dihydrate

**DOI:** 10.1107/S2414314623000962

**Published:** 2023-02-09

**Authors:** Ulises Aguinaga, Festus J. Oyinwola, Malik J. Childs, Sara Aldarkazanly, Muhammed Yousufuddin

**Affiliations:** a University of North Texas at Dallas, 7400 University Hills Blvd., Dallas, TX 75241, USA; Katholieke Universiteit Leuven, Belgium

**Keywords:** quaternary ammonium compound (QAC), benzalkonium chloride, BAC, crystal structure

## Abstract

The title compound, C_25_H_46_N^+^·Cl^−^·2H_2_O, crystallizes in the space group *P*2_1_ with one organic mol­ecule in the asymmetric unit. The compound belongs to a class of benzalkonium chlorides (BACs) with an alkyl chain length of 16 carbon atoms in an all-*trans* conformation.

## Abstract 2

The structure of a C_16_ benzalkonium chloride (C_16_BAC) is reported.

## Graphical abstract

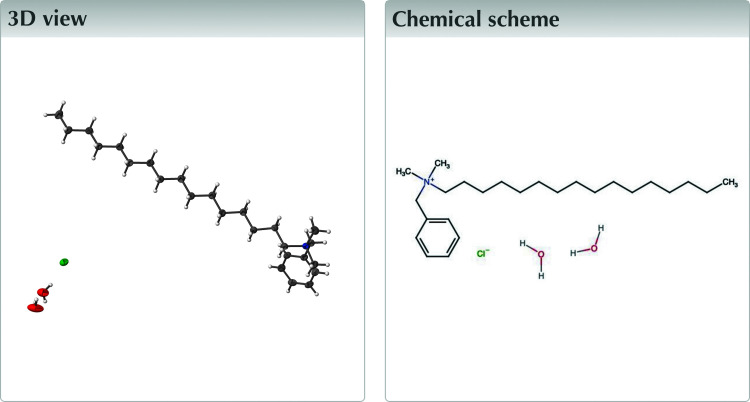

## Structure description

**Figure 1:**
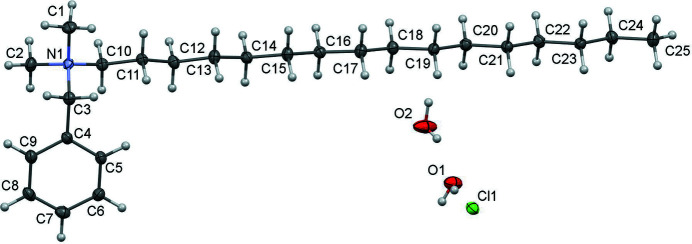
The title compound showing the atom-labelling scheme and 30% probability ellipsoids.

**Figure 2:**
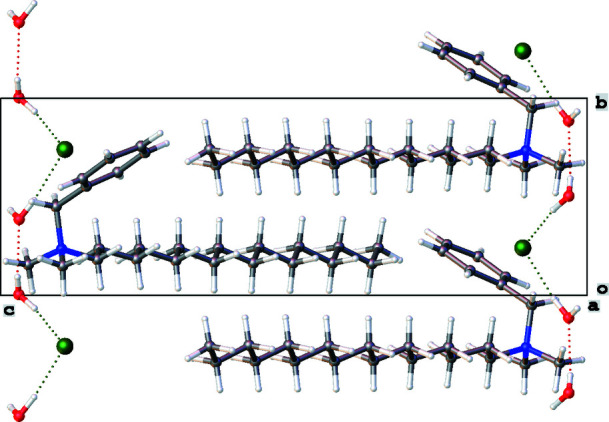
A view of the packing along the *a* axis showing the O—H⋯O and O—H⋯Cl hydrogen-bond inter­actions of the title compound.

Quaternary ammonium compounds (QACs) are one of the most visible and effective classes of disinfectants (Jennings *et al.*, 2015 ▸). The title compound, C_25_H_46_NCl·2H_2_O, crystallizes in the space group *P*2_1_ with one organic mol­ecule in the asymmetric unit (Fig. 1 ▸). The compound contains an alkyl group with a chain length of 16 carbon atoms in an all-*trans* conformation. It is therefore classified as a C_16_BAC (benzalkonium chloride). There are two water mol­ecules in the asymmetric unit making it a dihydrate. The oxygen atoms from each water mol­ecules are separated by a distance of 2.763 (5) Å and form together with the chloride ion a chain of hydrogen bonds running in the *b*-axis direction (Fig. 2 ▸, Table 1 ▸).

## Synthesis and crystallization

Benzylhexadecyldimethylammonium chloride was purchased from Sigma Aldrich and came in powder form. Crystals were grown from 95% ethanol solution at 253 K.

## Refinement

Crystal data, data collection and structure refinement details are summarized in Table 2 ▸.

## Supplementary Material

Crystal structure: contains datablock(s) I. DOI: 10.1107/S2414314623000962/vm4056sup1.cif

Structure factors: contains datablock(s) I. DOI: 10.1107/S2414314623000962/vm4056Isup2.hkl

Supporting information file. DOI: 10.1107/S2414314623000962/vm4056Isup3.mol

Supporting information file. DOI: 10.1107/S2414314623000962/vm4056Isup4.cml

CCDC reference: 2239551

Additional supporting information:  crystallographic information; 3D view; checkCIF report

## Figures and Tables

**Table 1:** Hydrogen-bond geometry (Å, °)

*D*—H⋯*A*	*D*—H	H⋯*A*	*D*⋯*A*	*D*—H⋯*A*
O1—H1*O*1⋯O2^i^	0.77 (4)	1.99 (4)	2.749 (5)	168 (4)
O2—H2*O*2⋯O1	0.71 (4)	2.09 (4)	2.763 (5)	160 (4)
O2—H1*O*2⋯Cl1^ii^	0.89 (4)	2.22 (4)	3.098 (4)	171 (4)
O1—H2*O*1⋯Cl1	0.89 (7)	2.34 (7)	3.210 (3)	167 (4)

Symmetry codes: (i) 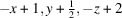 ; (ii) 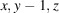 .

**Table 2:** Experimental details

Crystal data	
Chemical formula	C_25_H_46_N^+^·Cl^−^·2H_2_O
*M*_r_	432.11
Crystal system, space group	Monoclinic, *P*2_1_
Temperature (K)	167
*a*, *b*, *c* (Å)	9.051 (2), 7.0129 (17), 20.899 (5)
β (°)	92.388 (4)
*V* (Å^3^)	1325.4 (6)
*Z*	2
Radiation type	Mo *K*α
μ (mm^−1^)	0.16
Crystal size (mm)	0.15 × 0.10 × 0.02

Data collection	
Diffractometer	Bruker APEXII CCD
Absorption correction	Multi-scan (*SADABS*; Bruker, 2016 ▸)
*T*_min_, *T*_max_	0.674, 0.745
No. of measured, independent and observed [*I* > 2σ(*I*)] reflections	14073, 6583, 4958
*R*_int_	0.038
(sin θ/λ)_max_ (Å^−1^)	0.667

Refinement	
*R*[*F* ^2^ > 2σ(*F* ^2^)], *wR*(*F* ^2^), *S*	0.051, 0.125, 1.00
No. of reflections	6583
No. of parameters	281
No. of restraints	1
H-atom treatment	H atoms treated by a mixture of independent and constrained refinement
Δρ_max_, Δρ_min_ (e Å^−3^)	0.32, −0.22
Absolute structure	Flack *x* determined using 1730 quotients [(*I* ^+^)−(*I* ^−^)]/[(*I* ^+^)+(*I* ^−^)] (Parsons *et al.*, 2013 ▸)
Absolute structure parameter	0.03 (4)

Computer programs: *APEX2* and *SAINT* (Bruker, 2016 ▸), *SHELXT2014/4* (Sheldrick, 2015*a* ▸), *SHELXL2018/3* (Sheldrick, 2015*b* ▸) and *SHELXTL* (Sheldrick, 2008 ▸).

## full crystallographic data

### Crystal data

**Table:**

C_25_H_46_N^+^·Cl^−^·2H_2_O	*F*(000) = 480
*M**_r_* = 432.11	*D*_x_ = 1.083 Mg m^−^^3^
Monoclinic, *P*2_1_	Mo *K*α radiation, λ = 0.71073 Å
*a* = 9.051 (2) Å	Cell parameters from 2916 reflections
*b* = 7.0129 (17) Å	θ = 2.4–27.4°
*c* = 20.899 (5) Å	µ = 0.16 mm^−^^1^
β = 92.388 (4)°	*T* = 167 K
*V* = 1325.4 (6) Å^3^	Plate, colourless
*Z* = 2	0.15 × 0.10 × 0.02 mm

### Data collection

**Table:**

Bruker APEXII CCD diffractometer	4958 reflections with *I* > 2σ(*I*)
φ and ω scans	*R*_int_ = 0.038
Absorption correction: multi-scan (SADABS; Bruker, 2016)	θ_max_ = 28.3°, θ_min_ = 2.0°
*T*_min_ = 0.674, *T*_max_ = 0.745	*h* = −12→12
14073 measured reflections	*k* = −9→9
6583 independent reflections	*l* = −27→27

### Refinement

**Table:**

Refinement on *F*^2^	Hydrogen site location: mixed
Least-squares matrix: full	H atoms treated by a mixture of independent and constrained refinement
*R*[*F*^2^ > 2σ(*F*^2^)] = 0.051	*w* = 1/[σ^2^(*F*_o_^2^) + (0.060*P*)^2^] where *P* = (*F*_o_^2^ + 2*F*_c_^2^)/3
*wR*(*F*^2^) = 0.125	(Δ/σ)_max_ < 0.001
*S* = 1.00	Δρ_max_ = 0.32 e Å^−^^3^
6583 reflections	Δρ_min_ = −0.22 e Å^−^^3^
281 parameters	Absolute structure: Flack *x* determined using 1730 quotients [(*I*^+^)-(*I*^-^)]/[(*I*^+^)+(*I*^-^)] (Parsons *et al.*, 2013)
1 restraint	Absolute structure parameter: 0.03 (4)

### Special details

**Table:**

Geometry. All esds (except the esd in the dihedral angle between two l.s. planes) are estimated using the full covariance matrix. The cell esds are taken into account individually in the estimation of esds in distances, angles and torsion angles; correlations between esds in cell parameters are only used when they are defined by crystal symmetry. An approximate (isotropic) treatment of cell esds is used for estimating esds involving l.s. planes.
Refinement. The hydrogen atoms attached to oxygen in the water molecules were found in difference-Fourier maps and refined freely. Refinement was stable.

### Fractional atomic coordinates and isotropic or equivalent isotropic displacement parameters (Å^2^)

**Table:**

	*x*	*y*	*z*	*U*_iso_*/*U*_eq_	
Cl1	0.78448 (7)	0.74078 (14)	0.89033 (3)	0.0382 (2)	
N1	−0.1002 (2)	0.7343 (4)	0.10399 (9)	0.0263 (4)	
C1	−0.0259 (4)	0.6561 (5)	0.04714 (14)	0.0393 (7)	
H1A	−0.005079	0.520305	0.053854	0.059*	
H1B	0.066932	0.724714	0.041445	0.059*	
H1C	−0.090920	0.672073	0.008827	0.059*	
C2	−0.2521 (3)	0.6492 (5)	0.10567 (15)	0.0377 (7)	
H2A	−0.244371	0.509921	0.107298	0.057*	
H2B	−0.310118	0.687071	0.067117	0.057*	
H2C	−0.300958	0.695147	0.143706	0.057*	
C3	−0.1119 (3)	0.9502 (4)	0.09454 (13)	0.0296 (6)	
H3A	−0.010918	1.003024	0.091875	0.036*	
H3B	−0.165218	0.975446	0.053077	0.036*	
C4	−0.1892 (3)	1.0543 (4)	0.14620 (13)	0.0287 (6)	
C5	−0.1106 (3)	1.1249 (4)	0.19963 (14)	0.0323 (7)	
H5	−0.006986	1.104040	0.204155	0.039*	
C6	−0.1814 (3)	1.2248 (5)	0.24598 (13)	0.0351 (6)	
H6	−0.126969	1.271273	0.282577	0.042*	
C7	−0.3319 (3)	1.2573 (6)	0.23920 (14)	0.0383 (7)	
H7	−0.381005	1.322745	0.271954	0.046*	
C8	−0.4105 (3)	1.1963 (4)	0.18597 (15)	0.0384 (8)	
H8	−0.512972	1.224470	0.180730	0.046*	
C9	−0.3399 (3)	1.0929 (4)	0.13944 (15)	0.0355 (7)	
H9	−0.394892	1.048098	0.102750	0.043*	
C10	−0.0146 (3)	0.6807 (4)	0.16496 (12)	0.0281 (6)	
H10A	−0.062912	0.741108	0.201464	0.034*	
H10B	−0.021224	0.540895	0.170646	0.034*	
C11	0.1460 (3)	0.7366 (5)	0.16770 (11)	0.0271 (5)	
H11A	0.154701	0.875839	0.161056	0.032*	
H11B	0.197283	0.671417	0.132934	0.032*	
C12	0.2191 (3)	0.6831 (4)	0.23180 (13)	0.0310 (7)	
H12A	0.167339	0.748543	0.266354	0.037*	
H12B	0.209098	0.543991	0.238385	0.037*	
C13	0.3811 (3)	0.7359 (6)	0.23645 (12)	0.0326 (6)	
H13A	0.390185	0.875848	0.231735	0.039*	
H13B	0.431239	0.675867	0.200390	0.039*	
C14	0.4598 (3)	0.6761 (4)	0.29871 (13)	0.0306 (6)	
H14A	0.455246	0.535531	0.302471	0.037*	
H14B	0.407050	0.731138	0.334934	0.037*	
C15	0.6207 (3)	0.7384 (6)	0.30397 (12)	0.0333 (6)	
H15A	0.671767	0.690828	0.266170	0.040*	
H15B	0.624837	0.879488	0.303176	0.040*	
C16	0.7027 (3)	0.6675 (5)	0.36429 (13)	0.0313 (6)	
H16A	0.703514	0.526387	0.363884	0.038*	
H16B	0.648468	0.708978	0.402039	0.038*	
C17	0.8614 (3)	0.7392 (6)	0.37128 (12)	0.0335 (6)	
H17A	0.915067	0.700664	0.333038	0.040*	
H17B	0.860583	0.880332	0.372872	0.040*	
C18	0.9439 (3)	0.6639 (5)	0.43074 (14)	0.0329 (6)	
H18A	0.886678	0.695403	0.468689	0.040*	
H18B	0.949793	0.523191	0.427803	0.040*	
C19	1.0990 (3)	0.7434 (6)	0.44044 (12)	0.0310 (5)	
H19A	1.154957	0.716007	0.401789	0.037*	
H19B	1.092707	0.883721	0.444891	0.037*	
C20	1.1838 (3)	0.6637 (5)	0.49838 (14)	0.0317 (6)	
H20A	1.191804	0.523656	0.493579	0.038*	
H20B	1.127066	0.689056	0.536967	0.038*	
C21	1.3387 (3)	0.7467 (5)	0.50861 (12)	0.0300 (5)	
H21A	1.394675	0.723707	0.469660	0.036*	
H21B	1.330497	0.886361	0.514323	0.036*	
C22	1.4249 (3)	0.6639 (4)	0.56587 (14)	0.0316 (6)	
H22A	1.368587	0.686381	0.604758	0.038*	
H22B	1.433313	0.524313	0.560045	0.038*	
C23	1.5786 (3)	0.7463 (5)	0.57646 (12)	0.0302 (5)	
H23A	1.634517	0.726138	0.537323	0.036*	
H23B	1.570322	0.885506	0.583296	0.036*	
C24	1.6643 (3)	0.6597 (5)	0.63286 (15)	0.0365 (7)	
H24A	1.672148	0.520391	0.626087	0.044*	
H24B	1.608464	0.680219	0.672001	0.044*	
C25	1.8188 (3)	0.7413 (6)	0.64357 (15)	0.0438 (7)	
H25A	1.875770	0.719516	0.605374	0.066*	
H25B	1.868190	0.678556	0.680497	0.066*	
H25C	1.812198	0.878610	0.651734	0.066*	
O1	0.6599 (3)	0.3856 (4)	0.96854 (15)	0.0526 (7)	
O2	0.5853 (4)	0.0036 (5)	0.96896 (18)	0.0724 (11)	
H1O1	0.592 (4)	0.432 (6)	0.9830 (18)	0.052 (13)*	
H1O2	0.637 (4)	−0.066 (6)	0.9425 (19)	0.062 (12)*	
H2O2	0.623 (4)	0.092 (6)	0.9714 (19)	0.038 (12)*	
H2O1	0.681 (6)	0.482 (10)	0.943 (3)	0.12 (2)*	

### Atomic displacement parameters (Å^2^)

**Table:**

	*U*^11^	*U*^22^	*U*^33^	*U*^12^	*U*^13^	*U*^23^
Cl1	0.0299 (3)	0.0361 (4)	0.0489 (4)	0.0034 (4)	0.0046 (3)	−0.0021 (4)
N1	0.0242 (10)	0.0318 (11)	0.0230 (10)	−0.0031 (12)	0.0018 (8)	−0.0047 (12)
C1	0.0435 (18)	0.0447 (18)	0.0298 (15)	0.0023 (14)	0.0039 (13)	−0.0077 (13)
C2	0.0305 (16)	0.0390 (17)	0.0434 (17)	−0.0088 (13)	−0.0006 (13)	−0.0035 (14)
C3	0.0290 (14)	0.0306 (15)	0.0291 (14)	−0.0019 (11)	0.0003 (11)	0.0050 (12)
C4	0.0250 (13)	0.0261 (15)	0.0350 (15)	−0.0004 (11)	0.0027 (11)	0.0037 (12)
C5	0.0240 (14)	0.0355 (17)	0.0372 (16)	0.0005 (12)	0.0008 (12)	−0.0011 (13)
C6	0.0340 (14)	0.0340 (16)	0.0371 (14)	−0.0029 (15)	−0.0007 (11)	−0.0059 (16)
C7	0.0367 (14)	0.0365 (17)	0.0427 (16)	0.0018 (16)	0.0126 (12)	0.0003 (16)
C8	0.0243 (13)	0.037 (2)	0.0541 (18)	0.0038 (12)	0.0044 (13)	0.0051 (14)
C9	0.0291 (15)	0.0366 (17)	0.0406 (17)	−0.0009 (13)	−0.0028 (13)	0.0019 (14)
C10	0.0279 (13)	0.0324 (16)	0.0241 (13)	−0.0011 (11)	0.0026 (10)	0.0028 (11)
C11	0.0257 (11)	0.0301 (13)	0.0255 (12)	−0.0006 (15)	0.0027 (9)	0.0020 (14)
C12	0.0265 (13)	0.0373 (17)	0.0292 (14)	0.0010 (11)	0.0021 (11)	0.0051 (12)
C13	0.0281 (12)	0.0386 (15)	0.0309 (13)	−0.0041 (16)	−0.0017 (10)	0.0026 (16)
C14	0.0288 (14)	0.0323 (15)	0.0305 (14)	0.0037 (11)	−0.0004 (11)	0.0008 (12)
C15	0.0294 (13)	0.0363 (14)	0.0339 (13)	−0.0039 (16)	−0.0007 (10)	0.0033 (16)
C16	0.0289 (14)	0.0336 (15)	0.0315 (14)	0.0020 (12)	0.0006 (11)	0.0008 (12)
C17	0.0311 (13)	0.0377 (15)	0.0316 (13)	−0.0042 (17)	−0.0006 (10)	0.0018 (16)
C18	0.0296 (14)	0.0346 (15)	0.0346 (15)	−0.0022 (12)	0.0016 (12)	0.0013 (12)
C19	0.0282 (12)	0.0320 (14)	0.0328 (13)	−0.0038 (16)	−0.0006 (10)	−0.0025 (15)
C20	0.0288 (14)	0.0310 (14)	0.0353 (15)	−0.0010 (12)	0.0017 (12)	0.0009 (12)
C21	0.0291 (12)	0.0302 (13)	0.0305 (13)	−0.0010 (15)	0.0004 (10)	0.0002 (15)
C22	0.0288 (14)	0.0312 (15)	0.0348 (15)	−0.0008 (12)	0.0017 (12)	0.0032 (12)
C23	0.0288 (12)	0.0286 (13)	0.0333 (13)	−0.0012 (16)	0.0021 (10)	−0.0035 (15)
C24	0.0289 (15)	0.0382 (16)	0.0421 (17)	0.0003 (13)	−0.0014 (13)	0.0022 (14)
C25	0.0329 (14)	0.0498 (18)	0.0481 (17)	−0.002 (2)	−0.0078 (13)	−0.005 (2)
O1	0.0548 (18)	0.0417 (16)	0.0630 (18)	0.0013 (13)	0.0210 (14)	−0.0047 (13)
O2	0.077 (2)	0.0443 (18)	0.100 (3)	−0.0079 (17)	0.055 (2)	−0.0159 (18)

### Geometric parameters (Å, º)

**Table:**

N1—C1	1.494 (3)	C14—H14B	0.9900
N1—C2	1.500 (3)	C15—C16	1.520 (4)
N1—C10	1.511 (3)	C15—H15A	0.9900
N1—C3	1.530 (4)	C15—H15B	0.9900
C1—H1A	0.9800	C16—C17	1.523 (4)
C1—H1B	0.9800	C16—H16A	0.9900
C1—H1C	0.9800	C16—H16B	0.9900
C2—H2A	0.9800	C17—C18	1.518 (4)
C2—H2B	0.9800	C17—H17A	0.9900
C2—H2C	0.9800	C17—H17B	0.9900
C3—C4	1.500 (4)	C18—C19	1.517 (4)
C3—H3A	0.9900	C18—H18A	0.9900
C3—H3B	0.9900	C18—H18B	0.9900
C4—C5	1.390 (4)	C19—C20	1.514 (4)
C4—C9	1.391 (4)	C19—H19A	0.9900
C5—C6	1.375 (4)	C19—H19B	0.9900
C5—H5	0.9500	C20—C21	1.524 (4)
C6—C7	1.383 (4)	C20—H20A	0.9900
C6—H6	0.9500	C20—H20B	0.9900
C7—C8	1.364 (4)	C21—C22	1.517 (4)
C7—H7	0.9500	C21—H21A	0.9900
C8—C9	1.390 (4)	C21—H21B	0.9900
C8—H8	0.9500	C22—C23	1.513 (4)
C9—H9	0.9500	C22—H22A	0.9900
C10—C11	1.505 (4)	C22—H22B	0.9900
C10—H10A	0.9900	C23—C24	1.511 (4)
C10—H10B	0.9900	C23—H23A	0.9900
C11—C12	1.516 (4)	C23—H23B	0.9900
C11—H11A	0.9900	C24—C25	1.519 (4)
C11—H11B	0.9900	C24—H24A	0.9900
C12—C13	1.511 (4)	C24—H24B	0.9900
C12—H12A	0.9900	C25—H25A	0.9800
C12—H12B	0.9900	C25—H25B	0.9800
C13—C14	1.517 (4)	C25—H25C	0.9800
C13—H13A	0.9900	O1—H1O1	0.76 (4)
C13—H13B	0.9900	O1—H2O1	0.88 (7)
C14—C15	1.520 (4)	O2—H1O2	0.89 (4)
C14—H14A	0.9900	O2—H2O2	0.71 (4)
			
C1—N1—C2	108.4 (2)	C15—C14—H14B	108.9
C1—N1—C10	110.3 (2)	H14A—C14—H14B	107.7
C2—N1—C10	108.6 (2)	C16—C15—C14	113.5 (2)
C1—N1—C3	107.0 (2)	C16—C15—H15A	108.9
C2—N1—C3	109.8 (2)	C14—C15—H15A	108.9
C10—N1—C3	112.7 (2)	C16—C15—H15B	108.9
N1—C1—H1A	109.5	C14—C15—H15B	108.9
N1—C1—H1B	109.5	H15A—C15—H15B	107.7
H1A—C1—H1B	109.5	C15—C16—C17	113.4 (2)
N1—C1—H1C	109.5	C15—C16—H16A	108.9
H1A—C1—H1C	109.5	C17—C16—H16A	108.9
H1B—C1—H1C	109.5	C15—C16—H16B	108.9
N1—C2—H2A	109.5	C17—C16—H16B	108.9
N1—C2—H2B	109.5	H16A—C16—H16B	107.7
H2A—C2—H2B	109.5	C18—C17—C16	113.1 (2)
N1—C2—H2C	109.5	C18—C17—H17A	108.9
H2A—C2—H2C	109.5	C16—C17—H17A	108.9
H2B—C2—H2C	109.5	C18—C17—H17B	108.9
C4—C3—N1	114.9 (2)	C16—C17—H17B	108.9
C4—C3—H3A	108.6	H17A—C17—H17B	107.8
N1—C3—H3A	108.6	C19—C18—C17	113.7 (2)
C4—C3—H3B	108.6	C19—C18—H18A	108.8
N1—C3—H3B	108.6	C17—C18—H18A	108.8
H3A—C3—H3B	107.5	C19—C18—H18B	108.8
C5—C4—C9	118.6 (3)	C17—C18—H18B	108.8
C5—C4—C3	120.8 (2)	H18A—C18—H18B	107.7
C9—C4—C3	120.4 (3)	C20—C19—C18	114.0 (3)
C6—C5—C4	120.6 (3)	C20—C19—H19A	108.8
C6—C5—H5	119.7	C18—C19—H19A	108.8
C4—C5—H5	119.7	C20—C19—H19B	108.8
C5—C6—C7	119.9 (3)	C18—C19—H19B	108.8
C5—C6—H6	120.1	H19A—C19—H19B	107.6
C7—C6—H6	120.1	C19—C20—C21	113.8 (2)
C8—C7—C6	120.7 (3)	C19—C20—H20A	108.8
C8—C7—H7	119.7	C21—C20—H20A	108.8
C6—C7—H7	119.7	C19—C20—H20B	108.8
C7—C8—C9	119.6 (3)	C21—C20—H20B	108.8
C7—C8—H8	120.2	H20A—C20—H20B	107.7
C9—C8—H8	120.2	C22—C21—C20	113.9 (3)
C8—C9—C4	120.5 (3)	C22—C21—H21A	108.8
C8—C9—H9	119.8	C20—C21—H21A	108.8
C4—C9—H9	119.8	C22—C21—H21B	108.8
C11—C10—N1	115.4 (2)	C20—C21—H21B	108.8
C11—C10—H10A	108.4	H21A—C21—H21B	107.7
N1—C10—H10A	108.4	C23—C22—C21	114.2 (2)
C11—C10—H10B	108.4	C23—C22—H22A	108.7
N1—C10—H10B	108.4	C21—C22—H22A	108.7
H10A—C10—H10B	107.5	C23—C22—H22B	108.7
C10—C11—C12	110.8 (2)	C21—C22—H22B	108.7
C10—C11—H11A	109.5	H22A—C22—H22B	107.6
C12—C11—H11A	109.5	C24—C23—C22	113.6 (3)
C10—C11—H11B	109.5	C24—C23—H23A	108.9
C12—C11—H11B	109.5	C22—C23—H23A	108.9
H11A—C11—H11B	108.1	C24—C23—H23B	108.9
C13—C12—C11	112.5 (2)	C22—C23—H23B	108.9
C13—C12—H12A	109.1	H23A—C23—H23B	107.7
C11—C12—H12A	109.1	C23—C24—C25	113.8 (3)
C13—C12—H12B	109.1	C23—C24—H24A	108.8
C11—C12—H12B	109.1	C25—C24—H24A	108.8
H12A—C12—H12B	107.8	C23—C24—H24B	108.8
C12—C13—C14	114.0 (2)	C25—C24—H24B	108.8
C12—C13—H13A	108.7	H24A—C24—H24B	107.7
C14—C13—H13A	108.7	C24—C25—H25A	109.5
C12—C13—H13B	108.7	C24—C25—H25B	109.5
C14—C13—H13B	108.7	H25A—C25—H25B	109.5
H13A—C13—H13B	107.6	C24—C25—H25C	109.5
C13—C14—C15	113.4 (2)	H25A—C25—H25C	109.5
C13—C14—H14A	108.9	H25B—C25—H25C	109.5
C15—C14—H14A	108.9	H1O1—O1—H2O1	96 (5)
C13—C14—H14B	108.9	H1O2—O2—H2O2	105 (4)

### Hydrogen-bond geometry (Å, º)

**Table:**

*D*—H···*A*	*D*—H	H···*A*	*D*···*A*	*D*—H···*A*
O1—H1*O*1···O2^i^	0.77 (4)	1.99 (4)	2.749 (5)	168 (4)
O2—H2*O*2···O1	0.71 (4)	2.09 (4)	2.763 (5)	160 (4)
O2—H1*O*2···Cl1^ii^	0.89 (4)	2.22 (4)	3.098 (4)	171 (4)
O1—H2*O*1···Cl1	0.89 (7)	2.34 (7)	3.210 (3)	167 (4)

Symmetry codes: (i) −*x*+1, *y*+1/2, −*z*+2; (ii) *x*, *y*−1, *z*.

## References

[bb1] Bruker (2016). *APEX2*, *SAINT*, and *SADABS*. Bruker AXS Inc., Madison, Wisconsin, USA.

[bb6] Jennings, M. C., Minbiole, K. P. C. & Wuest, W. M. (2015). *ACS Infect. Dis.* **1**, 288–303. 10.1021/acsinfecdis.5b00047 27622819

[bb2] Parsons, S., Flack, H. D. & Wagner, T. (2013). *Acta Cryst.* B**69**, 249–259. 10.1107/S2052519213010014 PMC3661305 23719469

[bb3] Sheldrick, G. M. (2008). *Acta Cryst.* A**64**, 112–122. 10.1107/S0108767307043930 18156677

[bb4] Sheldrick, G. M. (2015*a*). *Acta Cryst.* A**71**, 3–8.

[bb5] Sheldrick, G. M. (2015*b*). *Acta Cryst.* C**71**, 3–8.

